# Attitudes and managing alcohol problems in general practice in Europe: results from the European ODHIN study

**DOI:** 10.1186/1940-0640-8-S1-A87

**Published:** 2013-09-04

**Authors:** Marcin Wojnar, Andrzej Jakubczyk, Peter Anderson, Ronda Gaby, Eileen Kaner, Marko Kolsek, Miranda Laurant, Dorothy Newbury-Birch, Cristina Ribeiro, Lidia Segura, Hana Sovinova, Fredrik Spak, Pierluigi Struzzo, Antoni Gual

**Affiliations:** 1 Department of Psychiatry, Medical University of Warsaw, Warsaw, Poland; 2 University of Newcastle, Newcastle-Upon-Tyne, UK; 3 Universiteit Maastricht, Maastricht, the Netherlands; 4 Univerza V Ljubljani, Lubljana, Slovenia; 5 Radboud University Nijmegen Medical Centre, Nijmegen, the Netherlands; 6 Instituto da droga e da toxicodepêndencia, I.P, Lisbon, Portugal; 7 Generalitat De Catalunya, Barcelona, Spain; 8 The National Institute of Public Health, Prague, Czech Republic; 9 Gotenborg Universitet, Gothenborg, Sweden; 10 Università degli studi di Udine, Udine, Italy; 11 Fundacio Privada Clinic per a la Recerca Biomèdica /Hospital Clinico i Provincial de Barcelona, Barcelona, Spain

## Aims

To assess and compare attitudes of general practitioners in different European countries towards screening and early interventions in alcohol use disorders.

## Methods

A total of 2435 general practitioners (GPs) from 9 European countries (Catalonia, Czech Republic, Italy, Netherlands, Poland, Portugal, Slovenia, Sweden and UK) were surveyed. The questionnaire included questions on demographic, education and training on alcohol, received by general practitioners, as well as their attitudes towards management of alcohol problems. In addition, the Shortened Alcohol and Alcohol Problems Perception Questionnaire (SAAPPQ) was used.

## Results

Seventy seven per cent of GPs declared that they placed ‘somewhat high’ or ‘very high’ their priority on disease prevention of the general practitioners; 54% reported having received 4 or more hours of education and training on managing alcohol problems, and 43% reported managing seven or more patients for alcohol problems in the previous year. GPs who reported higher levels of alcohol-related CME training were more likely to report regularly asking their patients about alcohol use (chi-square (3)=14.9, p=0.002). Moreover, there was a significant association between experience of alcohol-related CME and the number of patients managed for hazardous drinking (chi-square (5)=83.6, p<0.0005). Busyness (64%) and lack of training (52%) were considered most important barriers and readily available support services (84%) most effective facilitator of early intervention.

## Conclusion

The recommendations coming from the results of the ODHIN study for improving the delivery of early alcohol intervention and the management of alcohol problems in general practice are: provide better training and infrastructure. The main barriers and facilitators of early interventions did not change throughout last 16 years.

